# Initial High-Frequency Amplification Accuracy Predicts Long-Term Hearing Aid Fitting Outcomes: A Real Ear Measurement-Based Study

**DOI:** 10.3390/jcm15156081

**Published:** 2026-08-05

**Authors:** Chanhee Kim, Jinsei Jung

**Affiliations:** 1 Department of Otorhinolaryngology, Yonsei University College of Medicine, Seoul 03722, Republic of Korea; kchehdeod@yuhs.ac; 2 Won-Sang Lee Institute for Hearing Loss, Seoul 03722, Republic of Korea; 3 Severance Biomedical Science Institute, Yonsei University College of Medicine, Seoul 03722, Republic of Korea; 4 Bio-Centennial Convergence Institute, Yonsei University, Seoul 03722, Republic of Korea

**Keywords:** hearing aid, real ear measurement, target gain, hearing rehabilitation, amplification fitting, high frequency

## Abstract

**Background:** To investigate whether the accuracy of initial hearing aid amplification verified by real ear measurement (REM) predicts long-term fitting outcomes after one year, and to identify frequency-specific predictors associated with sustained fitting precision. **Methods:** This retrospective study included 426 ears from 269 patients who underwent initial REM during hearing aid fitting and repeat REM at 9–15 months after fitting. The primary outcome was the absolute difference between real ear insertion gain (REIG) and target gain (TG) for middle-level input sounds at one-year follow-up. Multivariable linear regression analysis was performed to evaluate demographic, audiologic, and initial REM-related predictors. **Results:** At initial fitting, the discrepancy between REIG and TG increased progressively toward higher frequencies, with the largest deviation observed at 6000 Hz (16.2 ± 9.5 dB). Multivariable analysis demonstrated that initial REM discrepancy at 6000 Hz was the strongest predictor of poorer fitting accuracy at one year (B = 0.129, *p* < 0.001). Initial discrepancies at 250 Hz and 500 Hz were also significant but showed weaker associations. Higher aided pure-tone thresholds and poorer aided word recognition scores were independently associated with less accurate long-term fitting outcomes. **Conclusions:** Initial amplification accuracy, particularly at high frequencies, plays a critical role in long-term hearing aid fitting stability. Achieving sufficient gain at 6000 Hz during the first fitting session may improve long-term fitting outcomes and reduce suboptimal hearing aid use.

## 1. Introduction

Hearing aids are the primary rehabilitation strategy for adults with sensorineural hearing loss (SNHL) [1]. Despite substantial advances in hearing aid technology and fitting algorithms, achieving an optimal fitting remains challenging in routine clinical practice [2]. Patients with similar audiograms often experience markedly different rehabilitation outcomes, and repeated fine-tuning is frequently required before acceptable listening comfort and speech clarity are achieved. Suboptimal initial fitting has been associated with lower patient satisfaction, reduced daily hearing aid use, and an increased likelihood of hearing aid abandonment, highlighting the importance of accurate fitting for successful hearing rehabilitation [3,4,5].

One of the major challenges during hearing aid fitting is determining whether the prescribed amplification accurately matches an individual’s acoustic requirements. Manufacturer first-fit algorithms frequently underestimate the gain required to achieve prescriptive targets, particularly at higher frequencies, resulting in insufficient amplification and poorer speech audibility [6]. Consequently, objective verification of hearing aid output has become an important component of evidence-based fitting.

Real-ear measurement (REM) is widely regarded as the gold standard for objectively verifying hearing aid fitting because it measures hearing aid output directly within the patient’s ear canal and allows comparison with prescriptive target gain. REM enables precise, input-level-specific gain adjustments for soft, medium, and loud sounds, thereby ensuring optimal audibility and comfort within the patient’s dynamic range [7,8]. Previous studies have consistently shown that REM improves the accuracy of hearing aid fitting compared with manufacturer first-fit settings [6,9,10,11,12,13]. Nevertheless, most previous investigations have focused on the immediate accuracy of gain matching or short-term patient outcomes, whereas little is known about whether the quality of the initial REM predicts long-term fitting outcomes [14,15].

Despite these established benefits, REM remains underutilized in clinical practice due to equipment costs and time constraints. Consequently, many patients receive suboptimal initial fittings based solely on first-fit algorithms. Unverified fitting often results in initial auditory discomfort, leading to low patient satisfaction, reduced daily wear time, or premature abandonment of hearing aids—a major contributor to the high rate of follow-up loss [5].

From a clinical perspective, identifying predictors of long-term fitting outcomes would be valuable because it could help clinicians recognize patients at risk of persistent fitting inaccuracy and prioritize additional follow-up or adjustment. However, it remains unclear which aspects of the initial REM are most strongly associated with long-term fitting success and whether frequency-specific fitting errors have different prognostic significance. Therefore, this study investigated whether the accuracy of the initial REM predicts long-term hearing aid fitting outcomes after one year. We further sought to identify frequency-specific REM parameters associated with sustained fitting accuracy.

## 2. Materials and Methods

### 2.1. Study Design

This retrospective study analyzed clinical data from patients who underwent REM as part of their hearing aid fitting process. During our initial data collection, a total of 324 patients were screened, of whom 55 were lost to follow-up, resulting in a follow-up loss rate of 17.0%. The remaining 269 patients (comprising 426 ears, accounting for bilateral fitting) were ultimately included in the final analysis. The inclusion criteria were as follows: (1) patients prescribed hearing aids who underwent an initial REM, and (2) patients who returned for a follow-up visit between 9 and 15 months post-initial fitting to undergo follow-up REM and subsequent re-fitting. Patients who did not meet this specific follow-up window or lacked complete REM data were excluded. This retrospective study was approved by the Institutional Review Board (IRB) of Yonsei University College of Medicine (IRB No. 4-2025-0892; approved on 26 August 2025). The requirement for written informed consent was waived because of the retrospective nature of the study. All procedures were conducted in accordance with the ethical principles of the Declaration of Helsinki.

### 2.2. Audiologic Assessment

Prior to HA fitting, all participants underwent comprehensive audiological evaluation, including pure-tone audiogram (PTA) and speech audiometry, to diagnose and establish baseline auditory function as previously reported [7,10,16]. The average of four frequencies (500, 1000, 2000, and 4000 Hz) presented in a pure-tone audiogram (PTA4) format was calculated and defined as the mean value. Hearing loss types were classified based on the PTA results. Specifically, the mean hearing threshold at low-to-mid frequencies (250, 500, and 1000 Hz) was subtracted from the mean threshold at high frequencies (2000, 4000, and 8000 Hz). A calculated value greater than 25 dB was defined as ski-slope type hearing loss, whereas a value of 25 dB or less was categorized as flat type.

The word recognition score (WRS) test was conducted for speech audiometry in a quiet environment using a phonetically balanced word list presented at the participants’ most comfortable listening level (MCL). The WRS was expressed as the percentage of correctly identified words, providing an objective measure of speech discrimination ability. The MCL was determined by gradually increasing the intensity of speech stimuli until the participant indicated the most comfortable loudness level for listening, as previously reported [7].

REM was performed to verify the accuracy of the HA fitting based on the NAL-NL2 fitting formula (Aurical, GN Otometrics, Taastrup, Denmark). Speech mapping was used to assess whether the amplified output of the HA matched the target gain using the input sound of an international speech test signal (ISTS) at the soft, moderate, and loud levels (45, 55, and 65 dB SPL, respectively). The REIG was measured to evaluate the difference between unaided and aided responses within the ear canal.

### 2.3. Statistical Analysis

To identify the predictors of long-term fitting accuracy, a multivariable linear regression analysis was conducted. Statistical analyses were performed using RStudio 2023.12.1+402. Linear regression assumptions were thoroughly verified. Multicollinearity was evaluated using Variance Inflation Factors (VIFs). To address residual non-normality and heteroscedasticity identified by Shapiro–Wilk and Breusch–Pagan tests, Heteroskedasticity-Consistent (HC3) robust standard errors were applied to obtain robust statistical inferences. The primary outcome was defined as the proximity of the Real Ear Insertion Gain (REIG) to the Target Gain (TG) at the one-year follow-up. Specifically, the outcome variable was the absolute difference between the REIG and TG (|REIG − TG|) measured during the one-year follow-up REM. A smaller value indicates a fitting that is closer to the prescribed target.

The independent variables incorporated into the regression model encompassed a comprehensive range of clinical and demographic factors. These included patient demographics such as age and sex, as well as baseline audiological profiles, specifically the mean Pure-Tone Audiometry (PTA) thresholds and Word Recognition Scores (WRS) in both unaided and aided conditions. We also accounted for the configuration of hearing loss, categorized into types such as ski-slope or flat, and the specific hearing aid style used by the patient (e.g., CIC, RIC, or BTE). Furthermore, to evaluate the influence of the initial fitting status, the absolute discrepancies between REIG and TG (|REIG − TG|) for moderate sound inputs at the initial REM were included as continuous variables across the frequency spectrum of 250, 500, 1000, 2000, 4000, and 6000 Hz. This multivariable approach allowed for a robust determination of which initial audiological and technical parameters significantly contribute to the long-term precision of hearing aid fitting.

## 3. Results

### 3.1. Participant Demographics

The demographic and clinical characteristics of the study population are summarized in Table 1. A total of 426 ears were included in the final analysis, with a mean patient age of 68.2 ± 14.2 years. The cohort consisted of 182 males (42.7%) and 244 females (57.3%). The mean unaided PTA threshold was 55.0 ± 12.3 dB HL, which improved to 46.2 ± 10.0 dB HL in the aided condition. Regarding the configuration of hearing loss, the flat type was more prevalent (*n* = 263, 61.7%) than the ski-slope type (*n* = 163, 38.3%). Receiver-in-canal (RIC) was the most common hearing aid type (*n* = 290, 68.1%), followed by completely in-canal (CIC) (*n* = 115, 27.0%). The mean unaided WRS was 58.2 ± 22.8%, showing an increase to 65.3 ± 20.7% with amplification.

Analysis of the initial REM data focused on the absolute discrepancy between the Real Ear Insertion Gain and the Target Gain (|REIG − TG|) for moderate sounds. At the initial fitting, the mean absolute difference across frequencies ranged from 7.0 to 16.2 dB. Notably, the gap between REIG and TG was significantly bigger at higher frequencies, specifically at 4000 Hz (12.4 ± 8.4 dB) and 6000 Hz (16.2 ± 9.5 dB), compared to lower and mid-frequencies. Furthermore, these high-frequency measurements exhibited larger standard deviations, indicating greater variability in achieving target gain in the high-frequency range during the initial fitting process.

### 3.2. Predictors of 1-Year Follow-Up Fitting Accuracy

A multivariable linear regression analysis was performed to identify factors significantly associated with the fitting outcome at the one-year follow-up (Table 2). The primary outcome was defined as the mean absolute difference between REIG and TG (|REIG − TG|) for moderate sounds at the one-year follow-up REM.

The regression model revealed that several audiological factors were significantly associated with the primary outcome. As hypothesized, mean aided WRS was a significant predictor; lower aided WRS was correlated with higher primary outcome values, suggesting poorer fitting precision after one year.

Most importantly, the study identified a significant relationship between initial REM indices and long-term fitting outcomes. Specifically, the initial |REIG − TG| at 6000 Hz for moderate sounds showed a strong positive correlation with the primary outcome (B = 0.129, *p* < 0.001). This indicates that a larger discrepancy from the target at 6000 Hz during the initial fitting is a statistically significant predictor of poor fitting accuracy one year later. Although statistically significant associations were also observed at 250 Hz (B = 0.134, *p* = 0.037) and 500 Hz (B = 0.132, *p* = 0.022), the *p*-values indicate that the initial REM result at 6000 Hz is the most robust predictor. Given the high variability (large standard deviation) observed at 6000 Hz in Table 1, these findings suggest that the initial fitting accuracy at this high-frequency marker serves as a critical indicator for predicting whether optimal fitting status can be maintained or achieved at the one-year follow-up.

**Figure 1:**
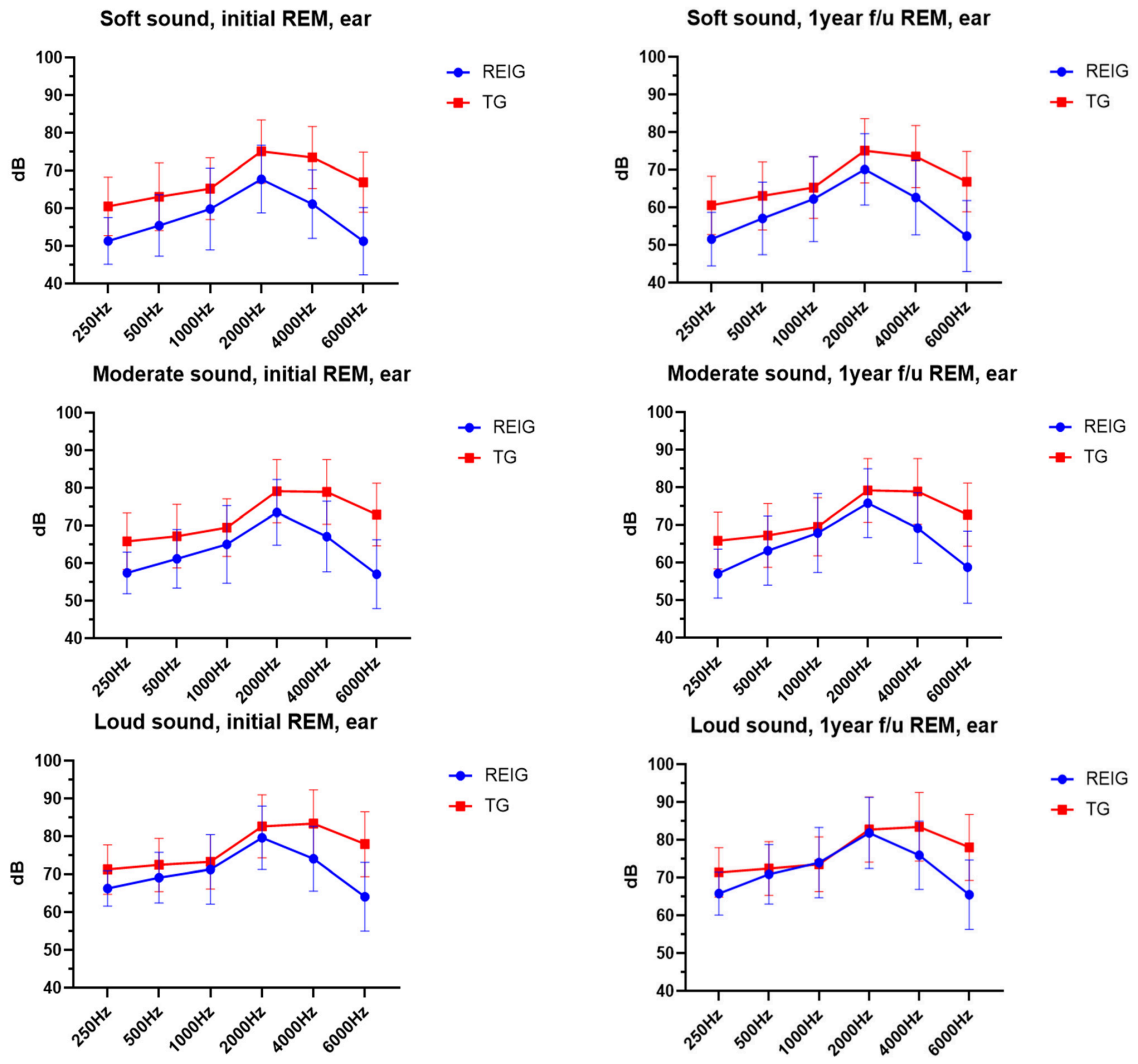
Comparison of real ear insertion gain (REIG) and target gain (TG) at initial fitting and 1-year follow-up across frequencies for soft (45 dB SPL), moderate (55 dB SPL), and loud (65 dB SPL) international speech test signal (ISTS) inputs.

Because the primary outcome of this study is expressed as a single value, relying solely on it limits the ability to evaluate longitudinal changes in REIG across the entire frequency spectrum during the 1-year follow-up. To address this, we compared REIG and TG at initial fitting with those at the 1-year follow-up across all frequencies under soft, moderate, and loud sound inputs (Figure 1). Notably, across all three input levels, the discrepancies between REIG and TG at 1000 Hz and 2000 Hz diminished over the 1-year follow-up period.

## 4. Discussion

REM is widely recognized as the reference standard for verifying hearing aid fitting because it objectively measures hearing aid output within the patient’s ear canal and quantifies the discrepancy between the real-ear insertion gain (REIG) and the prescribed target gain. Because manufacturer first-fit algorithms frequently fail to achieve prescriptive target gain, REM-based fitting is recommended to optimize amplification [6,13].

Previous studies have shown that fittings based solely on manufacturer first-fit algorithms frequently provide insufficient gain for soft sounds across both low- and high-frequency regions. In addition, high-frequency amplification often remains below prescribed targets even for loud input levels [12,17,18]. The relatively conservative gain prescribed at low frequencies may reflect an attempt to minimize upward spread of masking and improve overall listening comfort [19]. Conversely, insufficient high-frequency amplification is likely attributable to a conservative fitting strategy intended to reduce acoustic feedback, loudness discomfort, and patient rejection of amplified high-frequency sounds [20,21].

However, with recent advancements in hearing aid technology, some studies suggest that manufacturer-provided initial fits can now achieve gains comparable to REM-based fits [14,22]. A recent systematic review indicated that the evidence supporting the utility of REM varies from high to very low, and its efficacy may be diminished in patients with mild-to-moderate hearing loss [15]. Specifically, Caswell-Midwinter et al. reported that clinic-based fine-tuning—particularly those relying on immediate patient feedback—results in minimal gain adjustments and may not necessarily improve patient outcomes [23]. Similarly, Almufarrij et al. conducted a prospective study where patients experienced both initial fits and REM-based fits. Contrary to the conventional belief that REM is superior, patients tended to prefer the manufacturer’s initial fit, citing greater auditory comfort. Those who preferred the REM-based fit did so due to its greater clarity [24].

While the clinical significance of REM may seem diminished due to technological progress and issues of cost-effectiveness in clinical practice, REM remains vital for maximizing speech intelligibility. A limitation of the study by Almufarrij et al. was the lack of sufficient acclimatization time for the patients [24]. If interpreted conversely, this suggests that with adequate acclimatization, REM-based fitting can ultimately lead to superior clarity and speech intelligibility. Supporting this, the previous literature has shown that correcting the high-frequency gain deficiency common in manufacturer-only fittings can enhance speech discrimination, potentially exceeding the maximum speech-recognition scores measured during speech audiometry [17,25,26].

The present study addresses whether initial fittings that prioritize clarity over comfort yield long-term positive outcomes for patients. This research holds clinical significance as it analyzed fitting changes after a one-year follow-up period, providing ample time for sufficient acclimatization—a longitudinal perspective rarely found in existing REM literature. Our results indicate that a higher absolute difference between the REIG and Target Gain (REIG-TG) at 6000 Hz for middle sounds during the initial REM was associated with a higher mean absolute REIG-TG across all frequencies after one year. Additionally, insufficient gain at 250 and 500 Hz during the initial fitting significantly influenced gain deficiency at the one-year mark, second only to the 6000 Hz parameters. These findings suggest that prioritizing patient comfort by providing insufficient initial gain may adversely affect gain optimization across all frequencies in the long term. In clinical practice, particularly in patients who are highly sensitive or experience significant discomfort with sound amplification, it is difficult to provide sufficient initial gain at 6000 Hz. It is plausible that the final REIG in these specific patients remained low. Therefore, when performing REM and increasing gain, it is crucial to counsel patients on the trade-off between comfort and clarity and guide them toward settings that enhance speech intelligibility.

An important question raised by our findings is why the initial fitting accuracy at 6000 Hz, rather than at mid-frequencies, showed the strongest association with long-term fitting outcomes. One possible explanation is that high-frequency amplification represents the most challenging component of hearing aid fitting. High-frequency gain is frequently reduced during the initial fitting process because of concerns regarding acoustic feedback, loudness discomfort, and sound quality [12,13]. As a result, patients who receive insufficient high-frequency amplification at the initial fitting may continue to receive conservative gain adjustments during subsequent visits, leading to persistent under-amplification over time. Thus, the initial fitting strategy may establish the trajectory of subsequent fitting decisions rather than simply reflecting a single fitting session.

This study has several limitations. First, the primary outcome (dependent variable) may not fully represent patient satisfaction, as we could not incorporate survey results, such as the APHAB (Abbreviated Profile of Hearing Aid Benefit), due to inconsistent and incomplete data entries by patients. Second, the hearing aids included in this study were fitted between 2017 and 2020. Consequently, our findings may not fully reflect the technological advances in signal processing, feedback management, and automated fitting algorithms incorporated into the latest generations of hearing aids. Further prospective studies with larger, standardized cohorts are warranted to evaluate the independent effects of different hearing aid manufacturers and fitting algorithms on long-term fitting outcomes. Third, due to the retrospective nature of this study, we were unable to standardize the specific manufacturers or the internal fitting formulas used for each patient’s initial fit. It is possible that for some cases, there were inherent discrepancies between the manufacturer’s proprietary fitting rationale used during the initial setup and the NAL-NL2 prescriptive formula employed as the reference for REM. Finally, because patients lost to follow-up were excluded from the analysis, our study cohort may be subject to selection bias toward individuals with higher compliance and more successful rehabilitation outcomes.

## 5. Conclusions

In conclusion, the quality of the initial REM was significantly associated with long-term hearing aid fitting outcomes. Accurate gain matching, particularly at 6000 Hz and the low frequencies, may facilitate long-term fitting optimization. These findings support the continued clinical value of REM beyond immediate fitting verification.

## Figures and Tables

**Table 1:** Demographics of ears analyzed.

Factor	
Total analyzed ears, *n* (%)	426 (100)
Mean age ^a^ (SD)	68.2 (14.2)
Sex, *n* (%)	
Male	182 (42.7)
Female	244 (57.3)
Mean unaided PTA threshold ^b^ (SD), dB HL	55.0 (12.3)
Mean aided PTA threshold (SD), dB HL	46.2 (10.0)
Hearing loss type, *n* (%)	
Ski-slope type	163 (38.3)
Flat type	263 (61.7)
Hearing aid type, *n* (%)	
RIC type	290 (68.1)
CIC type	115 (27.0)
BTE type	5 (1.2)
ITC type	8 (1.9)
IIC type	3 (0.7)
Mean Word recognition score (SD), %	58.2 (22.8)
Mean aided Word recognition score (SD), %	65.3 (20.7)
Initial REM, middle sound, Mean |REIG − TG|, (SD), dB	
250 Hz	8.7 (6.2)
500 Hz	7.0 (5.5)
1000 Hz	7.0 (5.4)
2000 Hz	7.6 (5.7)
4000 Hz	12.4 (8.4)
6000 Hz	16.2 (9.5)

^a^ Age at first hearing aid fitting, ^b^ the mean value was calculated as the arithmetic mean of the hearing thresholds at 500 Hz, 1 kHz, 2 kHz, and 4 kHz.

**Table 2:** Multiple regression analysis for predicting 1-year follow-up absolute value of (REIG − TG).

Factor	B (Unstandardized)	SE	t-Value	*p*-Value
Age	0.019	0.016	1.164	0.245
Sex (Male)	0.649	0.405	1.601	0.110
Mean PTA threshold	0.047	0.038	1.241	0.215
Mean aided PTA threshold	0.060	0.033	1.796	0.073
Hearing loss type (Ski-slope)	0.694	0.429	1.618	0.106
Hearing aid type (RIC ref)				
BTE	−1.485	1.135	−1.307	0.191
CIC	−0.551	0.425	−1.297	0.195
IIC	−0.626	1.712	−0.365	0.714
ITC	−1.969	2.768	−0.711	0.477
Mean Word recognition score	−0.027	0.018	−1.475	0.140
Mean aided Word recognition score	0.046	0.021	2.190	0.029 *
Initial REM, middle sound, 250 Hz, |REIG − TG|	0.134	0.064	2.092	0.037 *
Initial REM, middle sound, 500 Hz, |REIG − TG|	0.132	0.057	2.294	0.022 *
Initial REM, middle sound, 1000 Hz, |REIG − TG|	0.059	0.044	1.350	0.177
Initial REM, middle sound, 2000 Hz, |REIG − TG|	−0.019	0.048	−0.396	0.692
Initial REM, middle sound, 4000 Hz, |REIG − TG|	−0.009	0.033	−0.282	0.777
Initial REM, middle sound, 6000 Hz, |REIG − TG|	0.129	0.036	3.533	<0.001 ***

*R*^2^ = 0.385, Adj.*R*^2^ = 0.359, *F* (17, 398) = 14.65. The mean value represents the average of the right and left ears. PTA_4_, Pure-tone average of 500, 1000, 2000, and 4000 Hz thresholds; BTE, Behind-The-Ear hearing aid; CIC, Complete-In-the-Canal hearing aid; RIC, Receive-In-the-Canal hearing aid; IIC, Invisible-In-the-Canal hearing aid; ITC, In-The-Canal hearing aid; Word Recognition Score, WRS; Real Ear Insertion Gain at the moderate sound level (55 dB SPL), REIG; Target Gain from NAL-NL2 formula, TG. * *p* < 0.05, *** *p* < 0.001. Model standard errors, t-values, and *p*-values were calculated using Heteroskedasticity-Consistent (HC3) robust standard errors to account for heteroscedasticity. Multicollinearity was confirmed to be minimal with all VIFs < 5 (range: 1.11–4.31).

## Data Availability

The original contributions presented in this study are included in the article. Further inquiries can be directed to the corresponding authors.
